# Methicillin-resistant *Staphylococcus aureus* (MRSA) infection of the temple region of the face: Case report

**DOI:** 10.1097/MD.0000000000042824

**Published:** 2025-06-13

**Authors:** Jennifer Sok Tin Ting, Ken Siong Hou Wong, Petrick Periyasamy, Roszalina Ramli

**Affiliations:** a Department of Oral and Maxillofacial Surgery, Hospital Canselor Tuanku Muhriz, Jalan Yaacob Latif, Kuala Lumpur, Malaysia; b Department of Oral and Maxillofacial Surgery, Faculty of Dentistry, Universiti Kebangsaan Malaysia, Jalan Raja Muda Abdul Aziz, Kuala Lumpur, Malaysia; c Department of Medicine, Faculty of Medicine, Universiti Kebangsaan Malaysia, Jalan Yaacob Latif, Kuala Lumpur, Malaysia.

**Keywords:** Bactrim, carbuncle, case report, methicillin-resistant *Staphylococcus aureus*, vancomycin

## Abstract

**Rationale:**

A carbuncle is a type of superficial soft-tissue infection involving hair follicles, typically starting as a furuncle. Multiple furuncles can coalesce to form a carbuncle. Methicillin-resistant *Staphylococcus aureus* (MRSA) is a common commensal organism on the skin and is known to cause infections, particularly in hospitalized patients or those living in nursing homes. This case report describes a patient with a right facial carbuncle complicated by co-infection with COVID-19 and drug toxicity.

**Patient concerns:**

A 73-year-old Malaysian Chinese woman with a history of chronic hypertension, diabetes mellitus, dyslipidemia, and cerebrovascular accident presented to the Emergency Department with dehydration, acute kidney injury, and a right temple carbuncle with pre-septal involvement.

**Diagnoses:**

A definitive diagnosis of the infection was made through a swab of the carbuncle pus, which was sent for microorganism culture and sensitivity testing.

**Interventions:**

At the Emergency Department, the patient was initially treated with intravenous (IV) ampicillin-sulbactam (Unasyn) 1.5 g 3 times daily. Despite this, the right facial swelling increased in size. On the fourth day of admission, IV metronidazole 500 mg 3 times daily was added, resulting in a notable reduction in swelling. Incision and drainage of the carbuncle were performed under local anesthesia, followed by daily wound dressings with diluted povidone-iodine solution. Based on blood culture and sensitivity results confirming MRSA infection, IV Unasyn and metronidazole were replaced with vancomycin 2 g initially, then adjusted to 1 g twice daily. However, vancomycin was discontinued after 3 days due to hyperkalemia, and oral Bactrim was initiated. The wound was dressed with hydrogen peroxide and povidone-iodine for their synergistic effects. The infection was effectively managed, leading to progressive wound healing.

**Outcomes:**

Complete healing of the MRSA-infected skin and soft tissue wound by secondary intention was achieved by the 7th week posthospital admission.

**Lessons:**

Localized MRSA infections of the face can be effectively managed with a combination of medical and surgical interventions. The defect in the temple area healed by secondary intention, resulting in an acceptable cosmetic outcome.

## 1. Introduction

A carbuncle is a type of superficial soft tissue infection involving hair follicles.^[1]^ It typically begins as a furuncle, which can progress to form an abscess.^[1]^ Multiple furuncles may coalesce into a larger infected mass characterized by multiple pus-draining points, thereby forming a carbuncle.^[2]^ Skin carbuncles are most commonly observed on the nape of the neck and the back,^[3]^ although several reports have described cases in the head and neck region.^[1,4,5]^ Carbuncles are frequently associated with immunocompromised patients and are recognized as one of the complications of diabetes mellitus.^[6]^

*Staphylococcus aureus* (*S aureus*) is a common pathogenic microorganism frequently responsible for various conditions, including endocarditis, bacteremia, osteomyelitis, and skin and soft tissue infections (SSTIs). SSTIs caused by *S aureus* exhibit a wide spectrum of severity, ranging from mild, self-limiting folliculitis to severe, life-threatening necrotizing soft tissue infections. Methicillin-resistant *S aureus* (MRSA), a known commensal organism on the skin, can cause infections influenced by factors such as the host’s immune response, pathogen virulence, and environmental conditions (e.g., surgical wounds).^[7]^ While most *S aureus*-related SSTIs are superficial and can be managed with oral antibiotics, they may occasionally progress to invasive infections necessitating parenteral therapy.^[8]^ In Malaysia, the prevalence of MRSA infections has been documented in several studies.^[9–11]^

SSTIs caused by *S aureus* have distinctive clinical features that facilitate differentiation from facial cellulitis secondary to odontogenic infections. *S aureus* infections commonly present as follicular infections, with clinical manifestations varying based on the depth and size of the infection. These may include folliculitis, furuncles (boils), or carbuncles.^[8]^ Although early *S aureus* infections typically begin superficially, they may spread into deeper tissues, potentially resulting in osteomyelitis or bacteremia. Despite advancements in antibiotic therapy, infection control measures, and active surveillance, MRSA remains a significant pathogen with persistently high morbidity and mortality rates.^[12,13]^

Intravenous (IV) vancomycin remains the drug of choice for hospital-acquired MRSA infections and is frequently used as both empirical and definitive therapy. The duration of antibiotic therapy for MRSA-related SSTIs ranges from 5 days to 2 weeks, depending on the patient’s clinical response. For patients unable to tolerate IV vancomycin, parenteral daptomycin is a viable alternative.^[14]^

## 2. Case report

**Figure 1.**
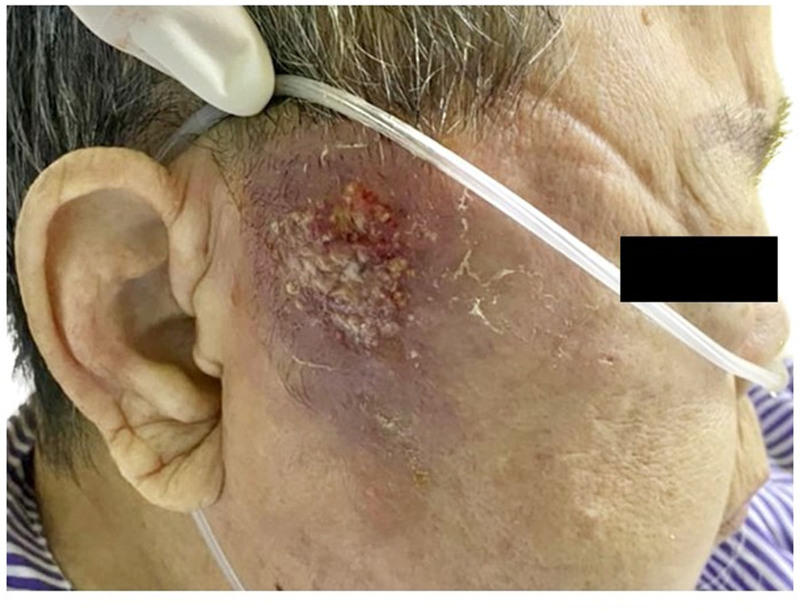
Carbuncles on the patient’s right face.

A 73-year-old Chinese woman was referred to the Emergency Department on March 28, 2022 with a two-week history of a right-sided facial swelling that began as a boil and progressed into a carbuncle (Fig. 1).

**Figure 2.**
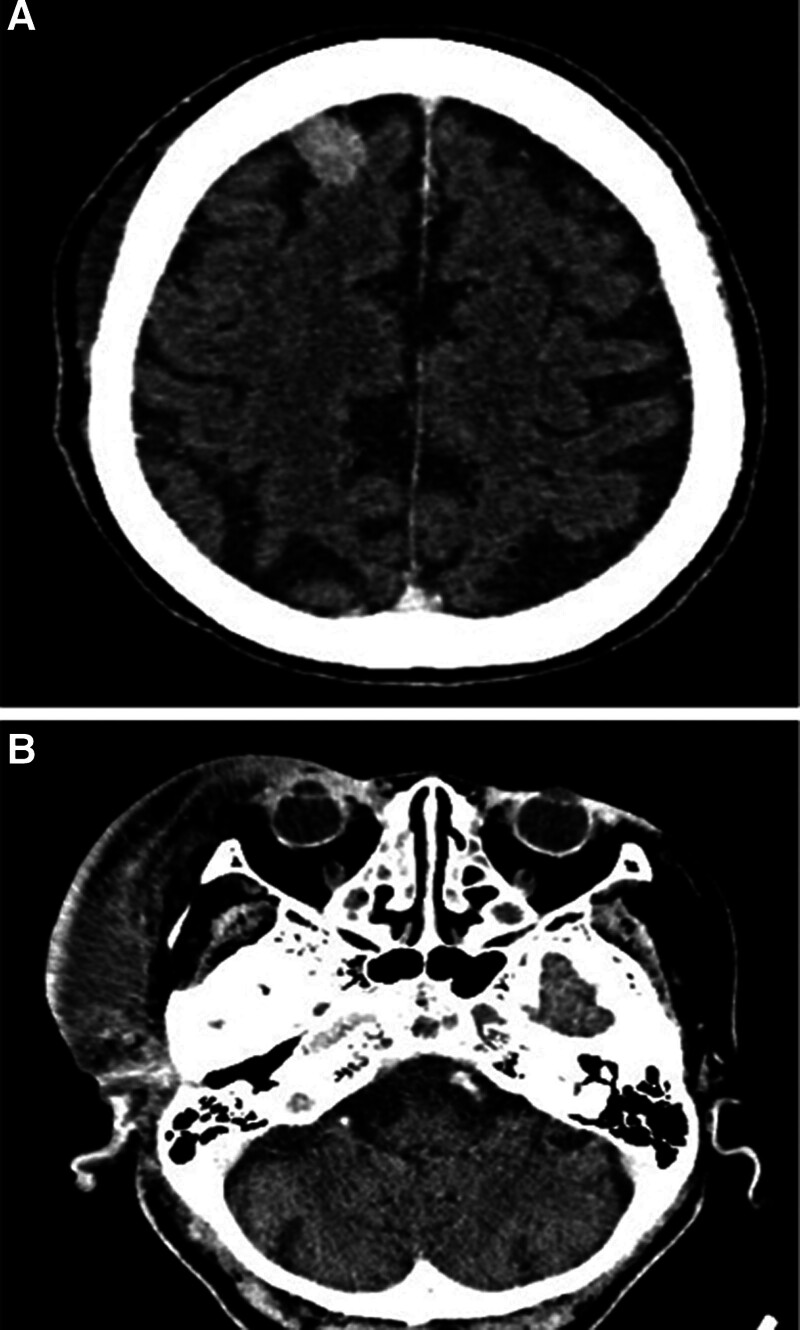
(A, B) CECT showed large right hemifacial and right scalp cellulitis with focal abscess at right temporal area. CECT = contrast-enhanced computed tomography.

The infection further complicated into an abscess with spreading cellulitis. Upon presentation, she had a reduced Glasgow Coma Scale of 10/15 and systemic toxicity, including fever (39.2°C), tachycardia (108 bpm), and hyperglycemia (17.1 mmol/L). Blood tests revealed anemia (Hb 9.2 g/dL), leukocytosis (white cell count [WCC] 28.7 × 10⁹/L), elevated C-reactive protein (12.04 mg/L), and serum creatinine (102.8 µmol/L). Contrast-enhanced CT showed extensive right hemifacial and scalp cellulitis with a focal abscess in the right temporal region (Fig. 2A and B). She was diagnosed with sepsis secondary to a facial carbuncle and acute kidney injury due to dehydration.

The patient tested positive for COVID-19 during routine preadmission screening and was subsequently admitted to the COVID ward. She had received 2 doses of the Pfizer vaccine, administered 8 and 9 months prior to her admission. Throughout her hospital stay, she remained asymptomatic. Daily assessments were conducted to monitor for any signs of COVID-19 infection; however, no treatment was required as she remained symptom-free.

Her medical history included hypertension, diabetes mellitus, dyslipidemia, and a cerebrovascular accident, managed with perindopril, amlodipine, atorvastatin, and insulin regimens. She resided in a nursing home under custodial care and was semi-independent in activities of daily living.

**Figure 3.**
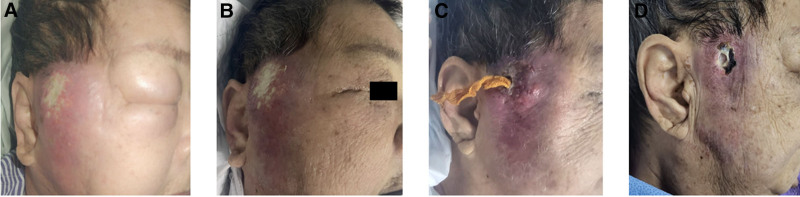
(A–D) The progress of the right temporal abscess secondary to infected carbuncle. (A) Worse facial appearance on Day 3 of admission. (B) Immediate reduction of her right temporal and pre-septal swelling was observed following administration iv metronidazole and correction of blood glucose. (C) Incision and drainage was performed under LA. Ribbon gauze soaked with diluted povidone-iodine packing was inserted into the cavity. (D) Wound was dry and no purulent discharge was observed.

Treatment commenced with IV Unasyn (ampicillin-sulbactam) 1.5 g 3 times daily (TDS). Due to poorly controlled hyperglycemia (27.8 mmol/L), her insulin regimen was adjusted to subcutaneous Insulatard 16 units twice daily and Actrapid 12 units TDS by the Endocrine Team. Daily wound dressing with diluted povidone-iodine (PVP-I) was initiated. On Day 3 of admission, her facial swelling worsened, and the diagnosis of a right temporal abscess with pre-septal involvement was confirmed (Fig. 3A). IV metronidazole 500 mg TDS was added on Day 4, resulting in a marked reduction in swelling later that day (Fig. 3B).

Incision and drainage (I&D) was performed on Day 5 under local anesthesia, with ribbon gauze soaked in diluted PVP-I packed into the cavity (Fig. 3C). Blood culture revealed MRSA, sensitive to vancomycin, Bactrim, and other antibiotics. After consultation with the Infectious Disease Team, IV vancomycin was initiated (2 g stat, followed by 1 g twice daily). However, vancomycin-induced hypokalemia (serum potassium 2.7 mmol/L) and an increasing WCC (29.9 × 10⁹/L) prompted a switch to oral Bactrim. Concurrent potassium supplementation (oral slow potassium 1.2 g TDS) normalized potassium levels within 3 days.

**Figure 4.**
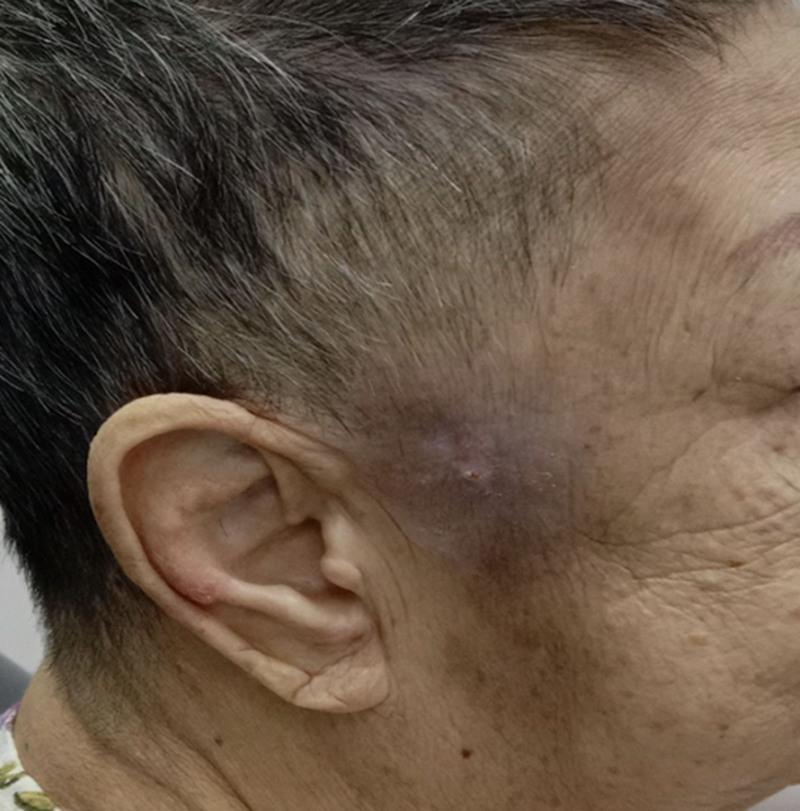
Complete healing was observed a month after hospital discharge.

By Day 15 (Day 6 of oral Bactrim), the wound showed no further discharge (Fig. 3D), and subsequent cultures were negative for MRSA. Initial plans for necrotic tissue excision and local flap reconstruction were revised as the wound decreased in size and showed significant improvement. Healing was allowed to progress by secondary intention. The patient was discharged on Day 21 with wound care instructions for her caregiver. At follow-up on Day 51, the wound had completely healed, leaving an acceptable cosmetic outcome (Fig. 4).

**Figure 5.**
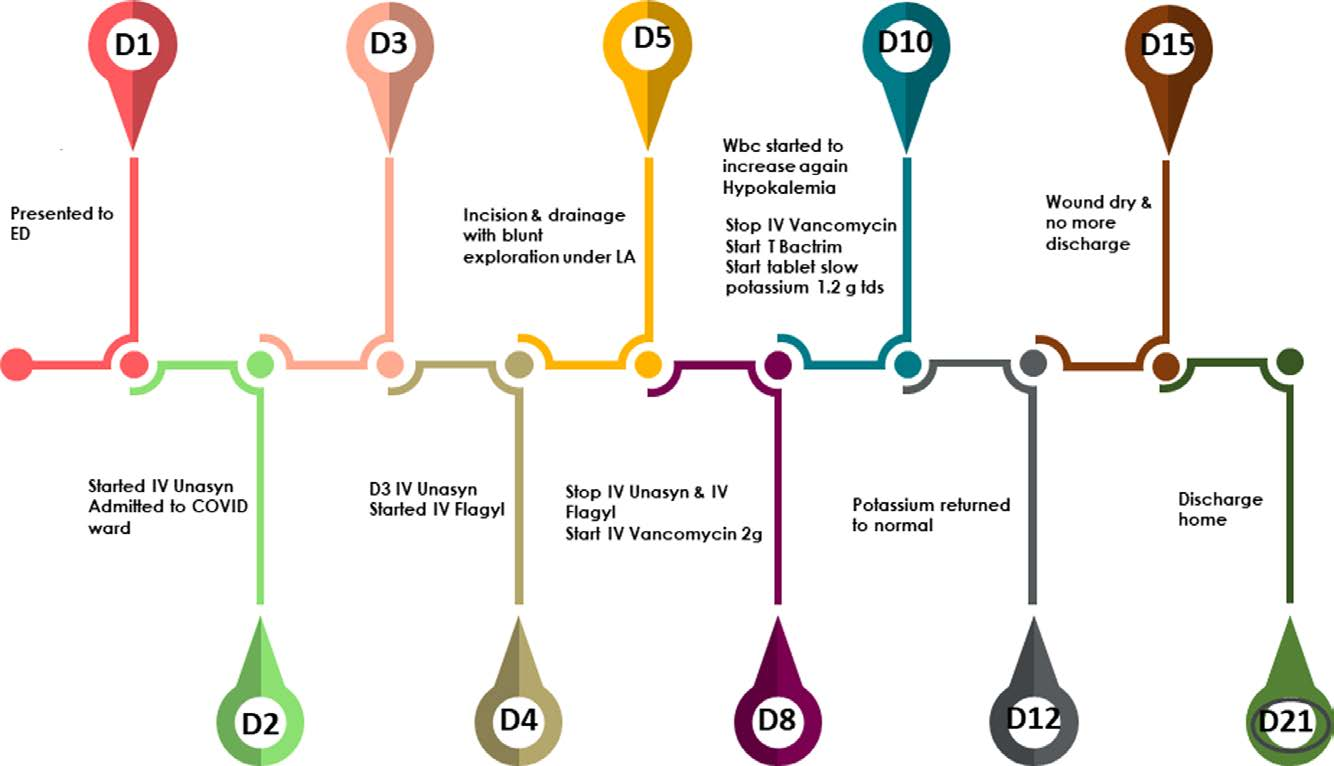
Timeline of key events during hospital admission.

For clarity, a summary of the clinical course is provided in Figure 5.

This study received approval from the Universiti Kebangsaan Malaysia Research Ethics Committee (UKM PPI/111/8/JEP-2022-090). Informed consent for publication was obtained from the patient.

## 3. Discussion

The World Health Organization has classified MRSA as a high-priority pathogen in need of new antibiotics, following a global increase in MRSA-related bloodstream infections, which rose from 21% in 2016 to 35% in 2020.^[15]^ MRSA is notorious for its resistance to nearly all classes of antibiotics.^[16]^ It carries toxin genes and pathogenicity islands that contribute to a wide range of illnesses, ranging from minor skin infections to life-threatening conditions such as septicemia and endocarditis.^[16]^

While necrotizing fasciitis, one of the most severe SSTIs, is classically associated with pathogens such as *Group A Streptococcus, Clostridium perfringens*, or mixed aerobic and anaerobic organisms, *S aureus* can also cause mono-microbial necrotizing fasciitis.^[8]^ The infection typically presents with local pain, along with systemic symptoms that may include fever or hypothermia, hypotension, and altered mental status.^[8]^ Early operative debridement is a key factor in determining the outcome of necrotizing fasciitis, making early recognition of this condition critical for achieving better therapeutic results. The Laboratory Risk Indicator for Necrotizing Fasciitis score is a reliable tool for detecting even early clinical cases of necrotizing fasciitis.^[17]^ The score uses routinely measured variables, such as total WCC, hemoglobin, sodium, glucose, serum creatinine, and C-reactive protein.^[17]^ Patients with a Laboratory Risk Indicator for Necrotizing Fasciitis score of 6 or higher should be carefully evaluated for necrotizing fasciitis.^[17]^ In this case, the patient scored 5, indicating a low risk for necrotizing soft tissue infections.

Evidence-based guidelines for the management of MRSA infections were developed by the Expert Panel of the Infectious Diseases Society of America.^[18]^ These guidelines are intended for use by healthcare providers managing adult and pediatric patients with MRSA infections.^[18]^ They cover the management of various clinical conditions associated with MRSA, including SSTIs.^[18]^ For cutaneous abscesses, I&D are the primary treatments.^[18]^ Antibiotic therapy is recommended in cases where abscesses are associated with severe or extensive disease (e.g., involving multiple sites of infection), rapid progression with accompanying cellulitis, signs and symptoms of systemic illness, underlying comorbidities or immunosuppression, extremes of age, abscesses in difficult-to-drain areas, associated septic phlebitis, or a lack of response to I&D alone.^[18]^

For hospitalized patients with complicated SSTI (cSSTI; defined as patients with deeper soft-tissue infections, surgical/ traumatic wound infection, major abscesses, cellulitis, and infected ulcers and burns), in addition to surgical debridement and broad-spectrum antibiotics, empirical therapy for MRSA should be considered, pending culture data.^[18]^ Options include the following: iv vancomycin, oral or iv linezolid 600 mg twice daily, iv daptomycin 4 mg/kg/dose once daily, iv telavancin 10 mg/kg/dose once daily, and iv clindamycin 600 mg or orally 3 times a day.^[18]^

Our patient was initially treated with Unasyn, a semisynthetic antibiotic ampicillin sodium and the beta-lactamase inhibitor sulbactam sodium for IV and intramuscular administration. Unasyn is not listed as one of the options for MRSA. The patient was started on IV vancomycin 2 g initially, followed by 1 g twice daily when we obtained the pus culture and sensitivity test which revealed MRSA on the eighth day of hospital admission. Vancomycin requires thorough monitoring for ensuring its therapeutic effectiveness and preventing nephrotoxicity as risk of acute kidney injury increases as vancomycin serum level increase. A thorough consensus review on the therapeutic monitoring of vancomycin is available from the American Society of Health-System Pharmacists, the Infectious Diseases Society of America, and the Society of Infectious Diseases Pharmacists.^[19]^

On the third day of vancomycin administration, a decline in serum potassium levels was observed in our patient, reaching its lowest point of 2.7 mmol/L. Vancomycin was immediately replaced with oral Bactrim, and tablet slow potassium 1.2 g 3 times a day was started. The potassium returned to normal value on the third day of vancomycin discontinuation, and second day of slow potassium administration. Bactrim exhibits good skin or soft tissue penetration.^[20]^ In a randomized controlled trial conducted by Talan et al (2016), the usage of Bactrim after drainage of cutaneous abscess showed a better prognosis than placebo in a group of patients with community-acquired MRSA.^[21]^

In general, vancomycin does not cause hypokalemia. However, there are reports that showed the association between vancomycin and hypokalemia.^[22–24]^ The hypokalemia episode induced by vancomycin in our patient could be explained based on the following clinical evidence. There was a clear association of drug exposure and the onset of the clinical condition. In addition, the serum potassium level returned to normal when vancomycin was discontinued.

All clinicians should be aware of this antimicrobial-related side effects as it can lead to a life-threatening complication.

Another inexplicable finding was related to metronidazole. Profound reduction of the right temple and pre-septal swelling was observed on the same day following IV metronidazole administration. Metronidazole is not listed as a first-line antibiotic of choice in managing MRSA infection. However, in the in-silico studies performed by Negi in 2016 and 2017, metronidazole-triazoles showed potential antibacterial effect against MRSA.^[25,26]^

In addition, the use of antiseptic irrigation and cleansing may contribute to antimicrobial activity. We used PVP-I and hydrogen peroxide as the antiseptic irrigation. PVP-1 was considered as a useful preoperative decolonizing agent for the prevention of *S aureus* infections, including MRSA and mupirocin-resistant strains.^[27]^

Hydrogen peroxide is an antiseptic and antibacterial agent that provides broad-spectrum efficacy against both gram-positive and gram-negative bacteria, bacterial spores, viruses, and yeast.^[28]^ It was shown that concurrent use of hydrogen peroxide and iodine leads to synergistic and additive inhibitory effects on bacteria and yeast. Combining these compounds often converts their individual static inhibitory effects into bactericidal effects.^[29]^

We decided to allow the wound to heal by secondary intention when we observed good progress during daily cleansing. The literature showed that wounds located on the concave surfaces of the face, for example, on the nose, eye, ear, and temple areas, usually heal with satisfactory functional and cosmetic outcomes.^[30,31]^ Healing process in this context depending on the wound contraction and it is influenced by the size of the wound, degree of surface concavity, adjacent skin laxity, and action of underlying skeletal muscles.^[32]^ A defect <2.5 cm, concave surface, superficial in nature and increased laxity, are all favorable factors for secondary healing.^[32,33]^ Our patient was 73 years old and the defect on her temple was about 2 cm and extended deep into the bone. Complete wound closure took about 51 days.

Healing by secondary intention, although a simple approach, may lead to decreased morbidity and, when used properly, may lead to better outcomes cosmetically and functionally, as compared with other more complicated procedures. It offers a quick, easy, and economical treatment for the properly selected patient. When used for superficial defects or defects located in concave areas of the face, the results can be as good as, or better than reconstruction with local flap or skin grafts.^[31–33]^

In the context of COVID-19 and MRSA infections, Habib et al. (2022) reported that MRSA was the most frequently identified pathogen among hospitalized COVID-19 patients, with a 5.52% rate of MRSA superinfection and a corresponding mortality rate of 25.23%.^[34]^ This observation is consistent with findings from a meta-analysis, which documented a 5.9% incidence of bacterial coinfections and a 14.3% rate of superinfections among COVID-19 patients.^[35]^ In our case, the patient remained asymptomatic throughout her hospital stay. She was initially quarantined in a COVID-19 ward and was subsequently transferred to the oral and maxillofacial surgery ward after 9 days for specialized management of her MRSA-infected facial wound. Notably, vaccinated individuals, particularly those aged 60 and above, have been shown to be more likely to remain asymptomatic.^[36]^

We were highly vigilant regarding the patient’s clinical condition, given her advanced age, poorly controlled diabetes mellitus, and concurrent MRSA and COVID-19 infections. Although the intensive care unit admission was considered and placed on standby, close monitoring and careful management allowed her to be successfully treated in the general ward.

## 4. Conclusion

MRSA infection frequently occurs in individuals with a history of hospitalization, residency in nursing facilities, or within community settings. Given its potential to cause severe complications and mortality, prompt diagnosis and tailored antibiotic therapy are crucial to preventing adverse outcomes. Additionally, as demonstrated in this case, the location of the defect allows for the use of noninvasive treatment options, offering the advantage of favorable cosmetic outcomes.

## Author contributions

**Conceptualization:** Roszalina Ramli.

**Data curation:** Jennifer Sok Tin Ting, Ken Siong Hou Wong.

**Methodology:** Jennifer Sok Tin Ting.

**Writing – original draft:** Jennifer Sok Tin Ting.

**Writing – review & editing:** Petrick Periyasamy, Roszalina Ramli.
